# Clinical Profiles and Survival Outcomes of Patients With Well-Differentiated Neuroendocrine Tumors at a Health Network in New South Wales, Australia: Retrospective Study

**DOI:** 10.2196/12849

**Published:** 2019-11-20

**Authors:** Jocelyn Reeders, Vivek Ashoka Menon, Anita Mani, Mathew George

**Affiliations:** 1 New South Wales Health Pathology Newcastle, New South Wales Australia; 2 New South Wales Health Pathology Sydney, New South Wales Australia; 3 New South Wales Health Pathology Tamworth, New South Wales Australia; 4 Hunter New England Area Health Service Tamworth Rural Referral Hospital Tamworth, New South Wales Australia

**Keywords:** Australia, neuroendocrine tumor, New South Wales

## Abstract

**Background:**

Neuroendocrine tumors (NETs) are a heterogeneous group of malignancies with varying and often indolent clinicobiological characteristics according to their primary location. NETs can affect any organ and hence present with nonspecific symptoms that can lead to a delay in diagnosis. The incidence of NETs is increasing in Australia; data regarding characteristics of NETs were collected from the cancer registry of Hunter New England, Australia.

**Objective:**

This study aimed to explore the clinical profiles and treatment and survival outcomes of patients with well-differentiated NETs in an Australian population.

**Methods:**

We reviewed the data of all adult patients who received the diagnosis of NET between 2008 and 2013. The clinicopathological, treatment, and follow-up data were extracted from the local Cancer Clinical Registry. We also recorded the level of remoteness for each patient by matching the patient’s residential postcode to the corresponding Australian Bureau of Statistics 2011 remoteness area category. Univariate analysis was used to find the factors associated with NET-related mortality. Survival analysis was computed.

**Results:**

Data from 96 patients were included in the study (men: 37/96, 38.5%, and women: 59/96, 61.5%). The median age at diagnosis was approximately 63 years. A higher proportion of patients lived in remote/rural areas (50/96, 52.1%) compared with those living in city/metropolitan regions (46/96, 47.9%). The most common primary tumor site was the gastroenteropancreatic tract, followed by the lung. The factors significantly associated with NET-related mortality were age, primary tumor site, surgical resection status, tumor grade, and clinical stage of the patient. At 5 years, the overall survival rate was found to be 62%, and the disease-free survival rate was 56.5%.

**Conclusions:**

Older age, advanced unresectable tumors, evidence of metastasis, and higher-grade tumors were associated with poorer outcomes. Lung tumors had a higher risk of NET-related mortality compared with other sites.

## Introduction

### Background

Neuroendocrine tumors (NETs) are a heterogeneous group of malignancies with often indolent clinicobiological characteristics with varying responses to therapy based on the primary tumor location and functional hormonal activity [1]. As these tumors arise from the neuroendocrine cells that are distributed throughout the body, almost any organ can be affected, including the lungs, small intestine, rectum, colon, appendix, and stomach [2,3]. This leads to various nonspecific symptoms and delay in diagnosis. Most NETs are indolent in nature, although some may proliferate rapidly and metastasize to other organs.

NETs were thought to be uncommon, accounting for approximately 2% of all malignant neoplasms; however, the incidence of NET is rising, as shown by different registries available [4,5].

The World Health Organization (WHO) in 2010 proposed a revised classification of NETs based on clinical, pathological, therapeutic, and prognostic factors, with an update released in 2017 [6,7]. Although the incidence of NETs appears to be increasing in Australia [8], the data of the characteristics of NETs among Australian patients are only starting to emerge [9]. Owing to the rarity and difficulty in diagnosis, the clinical, behavioral, and survival outcomes of patients with NETs in this demographic remain ill-defined.

### Objective

This retrospective analysis aimed to determine the incidence, clinical profile, and treatment and survival outcomes of rural and metropolitan patients with well-differentiated NETs in the Hunter New England area, New South Wales, Australia. The Hunter New England Local Health District covers a region of 131,785 square km. It encompasses a major metropolitan center (Newcastle) and regional communities (including Tamworth and Armidale), with a small percentage of people located in remote communities. The estimated resident population is 920,370 people [10].

## Methods

### Patients

Data were collected retrospectively from the local Cancer Clinical Registry, and all patients who received the diagnosis of NET (carcinoid, atypical carcinoid, and well-differentiated NET) between 2008 and 2013 were included. Hematoxylin- and eosin-stained slides that were available at our institution were reviewed for pathological diagnosis and grading according to the 2010 WHO classification and grading system as well as the updated recommendations in the 2017 WHO classification of endocrine organs [6,7]. As for slides that were unavailable for review, data were gathered from laboratory and clinical information systems. Gastroenteropancreatic (GEP) NETs were graded into 3 tiers (G1, G2, and G3) according to the following definitions of mitotic count and Ki-67 index: G1—mitotic count <2 per 10 high-power fields (HPFs) and/or <3% Ki-67 index, G2—mitotic count 2 to 20 per HPF and/or 3% to 20% Ki-67 index, and G3—mitotic coun*t* >20 per HPF and/or >20% Ki-67 index. Lung NETs were graded as G1 or typical carcinoid (carcinoid morphology and <2 mitoses/2 mm^2^, lacking necrosis) and G2 or atypical carcinoid (carcinoid morphology and 2-10 mitoses/2 mm^2^ or necrosis). Lung NETs with carcinoid morphology bu*t* >10 mitoses/2 mm^2^ were designated G3. NETs of an unknown primary site were graded based on the grading system of GEP NETs.

The mitotic index is based on the evaluation of mitoses in 50 HPFs (0.2 mm^2^ each) in areas of higher density and expressed as mitoses per 10 HPFs (2.0 mm^2^) [7]. The Ki-67 index was calculated using the MIB 1 antibody as a percentage of 500 to 2000 cells counted in areas of strongest nuclear labeling. When the grade differed for mitotic count and Ki-67 index for the same tumor, the higher of the two was taken [7]. Poorly differentiated neuroendocrine carcinomas at any site, and small-cell and large-cell neuroendocrine carcinomas of the lung were excluded because of their vastly different biological and survival profile.

Patient, tumor, treatment, and follow-up details were reviewed according to a predefined standard procedure. Patient characteristics included age at diagnosis, sex, and disease status at last follow-up. We also recorded the level of remoteness for each patient by matching the patient’s residential postcode to the corresponding Australian Bureau of Statistics (ABS) 2011 remoteness area (RA) category (2 groups were created: one representing regional Australia, ie, outer regional/inner regional/remote areas, and the other representing metropolitan areas, ie, major cities of Australia [11]). Furthermore, the Socio-Economic Indexes for Areas Index of Relative Socioeconomic Disadvantage (IRSD) was noted as an indicator of patient’s level of socioeconomic status [12]. The 2011 IRSD scores and deciles of the index were also recorded from the ABS website. Tumor characteristics included primary location (lung/gastrointestinal tract/pancreas/hepatobiliary system), size (<20 mm vs ≥20 mm), clinical stage (localized and regional vs distant and metastatic), grade, functional activity, and histology. Treatment characteristics included surgical procedures, somatostatin analogue therapy, or chemoradiation.

### Statistical Analysis

All statistical analyses were performed using SAS v9.4 (SAS Institute). The independent variables assessed in this study and included in all subsequent analyses were age, sex, cancer type, remoteness classification category, IRSD decile, tumor category, stage and grade of tumor at diagnosis, and receipt of resection surgery. Status of the patients was extracted from the records based on the last update. The main outcomes assessed in this study were all-cause and NET-related mortality. Furthermore, we also analyzed the 5-year overall survival (OS) and disease-free survival (DFS) rates. Kaplan–Meier analysis was used to estimate the cumulative OS rate. Crude hazard ratios (HRs) were calculated using Cox proportional hazards model to assess the factors associated with all-cause mortality. Competing risk regression model (Fine and Gray hazard model) was applied for assessing the factors associated with mortality because of NETs.

## Results

### Demographic Data

A total of 96 patients with NETs were included in this study (men: 37/96, 38.5%, and women: 59/96, 61.5%; male-to-female ratio, 1.0:1.5; age range, 25-101 years; and median age at diagnosis, 63 years [interquartile range, 51.5-72.5]). A total of 40 patients (40/96, 41.7%) were aged ≥65 years. A higher proportion of patients lived in the remote/rural areas (50/96, 52.1%) than in city-metropolitan areas (46/96, 47.9%). The demographic and clinicopathological details of all 96 patients of the study are described in Table 1.

**Table 1:** Characteristics of study participants and their distribution by cause of death.

Characteristic and category		Total (N=96), n (%)	Alive, n (%)^a^	Death due to a neuroendocrine tumor, n (%)^a^	Death due to other causes, n (%)^a^	*P* value
**Age at diagnosis (years)**						.001
	≤65	56 (58.3)	43 (76.8)	8 (14.3)	5 (8.9)	
	>65	40 (41.7)	17 (42.5)	9 (22.5)	14 (35.0)	
**Sex**						.61
	Male	37 (38.5)	21 (56.8)	7 (18.9)	9 (24.3)	
	Female	59 (61.5)	39 (66.0)	10 (17.0)	10 (17.0)	
**Neuroendocrine tumor site**						.002
	Lung	30 (31.3)	15 (50.0)	10 (33.3)	5 (16.7)	
	Gastroenteropancreatic	55 (57.3)	42 (76.4)	3 (5.5)	10 (18.1)	
	Other^b^	11 (11.5)	3 (27.2)	4 (36.4)	4 (36.4)	
**Grade at diagnosis^c^**						.01
	1	46 (74.2)	35 (76.1)	2 (4.4)	9 (19.5)	
	2-3	16 (25.8)	9 (56.3)	5 (31.2)	2 (12.5)	
**Index of relative socioeconomic disadvantage category**						.30
	<5	54 (56.3)	37 (68.5)	7 (13.0)	10 (18.5)	
	≥5	42 (43.8)	23 (54.8)	10 (23.8)	9 (21.4)	
**Remoteness category**						.22
	City	46 (47.9)	25 (54.4)	11 (23.9)	10 (21.7)	
	Regional/remote	50 (52.1)	35 (70.0)	6 (12.0)	9 (18.0)	
**Resection surgery^c^**						<.001
	No	30 (31.3)	10 (33.3)	13 (43.3)	7 (23.4)	
	Yes	65 (67.7)	49 (75.4)	4 (6.2)	12 (18.4)	
**Stage at diagnosis^c^**						.01
	Localized	40 (41.7)	32 (80.0)	1 (2.5)	7 (17.5)	
	Regional	25 (26.0)	15 (60.0)	5 (20.0)	5 (20.0)	
	Distant	30 (31.3)	13 (43.3)	11 (36.7)	6 (20.0)	
**Tumor size category^c^**						.47
	<20 mm	33 (47.8)	24 (72.7)	3 (9.1)	6 (18.2)	
	≥20 mm	36 (52.2)	23 (63.9)	7 (19.4)	6 (16.7)	

^a^In these columns, the percentage values within parentheses have been calculated row-wise, for example, for the third row, (43/56)×100=76.8 where N is 56.

^b^Other sites include anterior mediastinum (n=1), ovary (n=1), retroperitoneum (n=1), and unknown primary (n=8).

^c^Information on grade was missing at diagnosis for 34 patients, on resection surgery for 1 patient, on stage at diagnosis for 1 patient, and on tumor size for 27 patients.

Of the total 96 patients, 36 (36/96, 37.5%) died during follow-up (17/96, 18% because of disease and, 19/96, 20% because of other or unknown causes). The number of deaths was greater among men (16/37, 43%) than among women (20/59, 33.9%).

### Clinicopathological Data

The most common primary site was the GEP tract (55/96, 57.3%), followed by the lung (30/96, 31.3%), and others (11/96, 11.5%). Of the total 96 patients, 35 (35/96, 36.4%) had functional tumors causing carcinoid syndrome. Distant metastases were observed in 30 patients (30/96, 31.3%); 25 patients (25/96, 26%) had regional spread of disease and 40 (40/96, 41.7%) had localized disease. In patients with metastases, metastases to the liver were the most common (27/30, 90%). Overall, 33 patients (33/96, 34.4%) had a tumor size of <20 mm and 36 (36/96, 37.5%) had a tumor size of ≥20 mm; data of the remaining 27 patients were not available. In total, 46 patients (46/96, 47.9%) had grade 1, 12 (12/96, 12.5%) had grade 2, 4 (4/96, 4.2%) had grade 3, and 34 (34/96, 35.4%) had an unknown grade.

### Survival and Prognostic Factors

**Figure 1:**
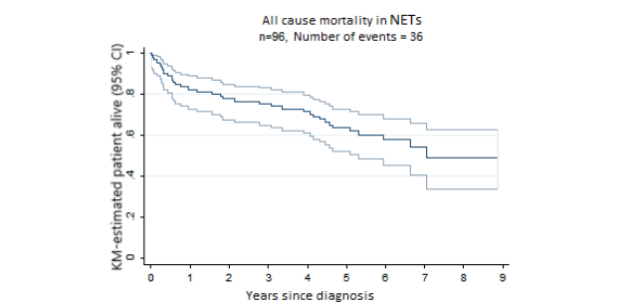
Kaplan–Meier (KM) curves for all-cause mortality in patients with neuroendocrine tumors (NETs). Median overall survival (50th percentile) was 7.04 years and 5-year overall survival was 62%.

**Figure 2:**
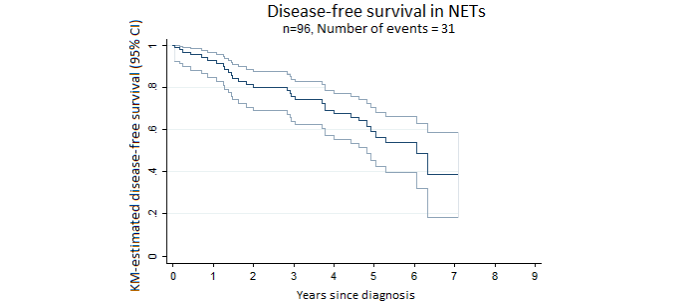
Disease-free survival (DFS) in patients with neuroendocrine tumors (NETs). Median DFS (50th percentile) was 6.04 years and 5-year DFS at 5 years was 56.5%. KM: Kaplan–Meier.

**Figure 3:**
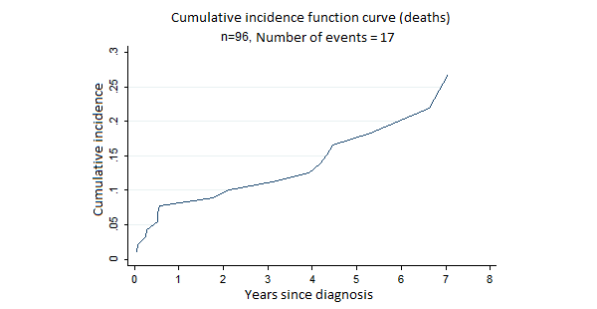
Cumulative incidence function curve for neuroendocrine tumors–related mortality, with other causes of mortality included as a competing risk.

Most patients (65/96, 67.7%) underwent resection surgery. The median (interquartile range) follow-up was 4.6 (1.03-5.91) years. The median OS period was 7.04 years and median DFS, 6.04 years. Overall 5-year survival (OS) rate was 62% (Figure 1). The 5-year DFS rate was 56.5% (Figure 2). Figure 3 shows the incidence curve for neuroendocrine cancer–related mortality, having other causes of mortality included as a competing risk.

### Patient Characteristics Associated With All-Cause Mortality

Table 2 lists all factors significantly associated with all-cause mortality at univariate level.

Older age was found to be significantly associated with an increased risk of mortality (HR 3.05, 95% CI 1.54-6.06; *P*=.001). Men had significantly higher HRs than women, suggesting an increased risk of all-cause mortality among men (HR 4.33, 95% CI 1.52-12.37; *P*=.02). Patients with GEP NETs had a lower risk of mortality compared with those with NETs of other or unknown sites (HR 0.25, 95% CI 0.10-0.61; *P*=.002). However, there was no difference in the risk of cancer-related mortality between those with GEP NETs and NETs of other or unknown sites. Those who had not received resection surgery had a higher risk of experiencing all-cause mortality than those who had received resection surgery (HR 3.25, 95% CI 1.68-6.30; *P*<.001). Patients with distant metastases had a higher risk of experiencing all-cause mortality than those with a localized or regional tumor (HR 2.15, 95% CI 1.10-4.18; *P*=.02).

**Table 2:** Crude hazard ratios based on Cox proportional hazards model to assess characteristics associated with all-cause mortality in patients with neuroendocrine tumors (N=96).

Characteristic^a^ and category		Total deaths (N)	Crude hazard ratio^b^ (95% CI)	*P* value
**Age at diagnosis (years)**				
	≤65	13	Reference	
	>65	23	3.05 (1.54-6.06)	.001
**Sex**				
	Male	16	4.33 (1.51-12.37)^c^	.01
	Female	20	Reference	
**Neuroendocrine tumor site**				
	Lung	15	0.89 (0.37-2.13)	.80
	Gastroenteropancreatic	13	0.25 (0.10-0.61)	.002
	Other	8	Reference	
**Grade at diagnosis**				
	1	11	Reference	
	2-3	7	1.81 (0.69-4.72)	.23
**Remoteness category**				
	City	21	1.56 (0.80-3.03)	.19
	Regional/remote	15	Reference	
**Resection surgery**				
	No	20	3.25 (1.68-6.30)	.001
	Yes	16	Reference	
**Stage at diagnosis**				
	Localized/regional	18	Reference	
	Distant	17	2.15 (1.10-4.18)	.03
**Tumor size category**				
	<20 mm	9	Reference	
	≥20 mm	13	1.43 (0.61-3.36)	.41

^a^Information on grade was missing at diagnosis for 34 patients, on resection surgery for 1 patient, on stage at diagnosis for 1 patient, and on tumor size for 27 patients.

^b^Hazard ratios were based on Cox proportional hazards model.

^c^Hazard ratio adjusted for time interaction.

### Patient Characteristics Associated With Neuroendocrine Tumors–Related Mortality

Table 3 lists all factors significantly associated with NET-related mortality at the univariate level.

Patients with GEP NETs had a lower risk of mortality compared with those with NETs of other or unknown sites (HR 1.41, 95% CI 0.44-4.52; *P*=.01). However, there was no difference in the risk of cancer-related mortality between those with lung NETs and NETs of other or unknown sites. Those who had not received resection surgery had a higher risk of experiencing all-cause and cancer-related mortality than those who had received resection surgery (HR 35.3, 95% CI 7.75-160.82; *P*=.001). Patients with NETs staged as distant had a higher risk of experiencing NET-related mortality than those with a localized or regional tumor (HR 3.93, 95% CI 1.44-10.68; *P*=.01). Patients diagnosed with a grade 2/3 tumor had a higher risk of experiencing cancer-related mortality than those diagnosed with a grade 1 tumor (HR 6.83, 95% CI 1.38–33.75; *P*=.02).

**Table 3:** Crude hazard ratios based on competing risk regression model to assess characteristics associated with neuroendocrine tumors–related mortality (N=96).

Characteristic and category^a^		Deaths (N)	Crude hazard ratio^b^ (95% CI)	*P* value
**Age at diagnosis (years)**				
	≤65	8	Reference	
	>65	9	1.57 (0.62-4.01)	.34
**Sex**				
	Male	7	1.08 (0.41-2.86)	.87
	Female	10	Reference	
**Neuroendocrine tumor site**				
	Lung	10	1.41 (0.44-4.55)	.01
	Gastroenteropancreatic	3	0.14 (0.03-0.63)	.56
	Other	4	Reference	
**Grade at diagnosis**				
	1	2	Reference	
	2/3	5	6.83 (1.36-34.21)	.02
**Remoteness category**				
	City	11	1.89 (0.70-5.13)	.21
	Regional/remote	6	Reference	
**Resection surgery**				
	No	13	35.31 (7.69-162.2)^c^	.001
	Yes	4	Reference	
**Stage at diagnosis**				
	Localized/regional	6	Reference	
	Distant	11	3.93 (1.44-10.74)	.01
**Tumor size category**				
	<20 mm	3	Reference	
	≥20 mm	7	2.19 (0.57-8.49)	.26

^a^Information on grade was missing at diagnosis for 34 patients, on resection surgery for 1 patient, on stage at diagnosis for 1 patient, and on tumor size for 27 patients.

^b^Hazard ratio was based on the competing risk regression model (Fine and Gray hazard model), and “death due to other causes” was considered a competing risk.

^c^Hazard ratio adjusted for time interaction.

## Discussion

### Principal Findings

NETs originate from neuroendocrine cells, with the most common sites being the small bowel, rectum, appendix, colon, stomach, and lungs. Nevertheless, NETs can arise in almost any organ. In this retrospective study, we collected and analyzed the incidence, clinical profiles, and treatment outcomes of 96 patients with low-grade NETs over a 5-year duration. The patient characteristics significantly associated with death due to NETs were older age, tumor type, stage at diagnosis, and grade at diagnosis.

### Limitations

Our study had a few limitations. Multivariate analyses were precluded by the limited patient population; hence, we have only presented unadjusted analyses of our findings. Therefore, evaluation in a larger population of such tumors is warranted.

### Comparison With Previous Studies

The gastrointestinal tract is believed to be the most frequent location of NETs—confirmed by our data of an Australian population—followed by the lung and others. Our results confirm and corroborate findings reported in the epidemiological study by Luke et al [9]. However, another recent study involving advanced NETs has demonstrated the small intestine to be the most common site, closely followed by the lung [13]. An analysis of our study results revealed that the primary site of the tumor is a major factor associated with mortality, as patients with GEP NETs had a significantly lower risk compared with those with lung NETs, extraintestinal NETs, and NETs of an unknown primary site.

The median age of patients at diagnosis in our study was approximately 63 years. This finding is similar to that reported in a study in the United States where the median age at diagnosis was 63 years [4]. Our results show that older age is significantly associated with an increased risk of both all-cause and NET-related mortality. This is in line with the findings of the study by Strosberg and Cheema [14], who had evaluated the data of 425 patients with pancreatic NETs.

Previous data have shown that survival in patients with NETs varies according to the tumor grade, and hence it is an important factor to predict survival. The American Joint Committee on Cancer reported an HR of 2.3 in intermediate-grade tumors versus low-grade tumors and of 5.4 in high-grade tumors versus low-grade tumors [14]. Our results found that patients with grade 2/3 tumors had a higher risk of experiencing cancer-related mortality than those with a grade 1 tumor.

In accordance with other studies in different geographic regions, metastatic disease at diagnosis and higher grade of tumors were associated with mortality. The rate of distant metastases in our series (31.3%) was slightly higher compared with that reported by Taal and Visser in their study (12%-25%) [15]. This could be explained by the higher proportion of patients living in remote/rural areas in our study population, which could be attributed to poorer access to advanced health care services. This finding is in line with that from a previous study [14]. In addition, tumors of unknown primary (n=8) were included in this study, which might have resulted in a bias toward a higher rate of distant metastases in our series.

Nearly one-third of the Australian population live in regional and remote areas, and the proportion of cancer-related deaths is observed to be higher in this demographic [16]. For both sexes, the age-standardized mortality rates of the regional and rural areas have shown no evidence of improvement as opposed to that among the urban residents [17]. This is plausibly related to the access to specialized cancer care in addition to other factors such as higher prevalence of cancer risk factors, such as smoking and sun exposure, and higher prevalence of other comorbidities.

### Conclusions

In our cohort of patients with NETs from rural and metropolitan regions of Australia, we have shown that older age, extraintestinal NETs, unresectable tumors, evidence of metastasis, and higher-grade tumors contributed to significantly poorer outcomes. Furthermore, patients from rural/remote areas have inferior clinical outcomes compared with those from city/metropolitan areas.

## Abbreviations

ABS: Australian Bureau of Statistics
DFS: disease-free survival
GEP: gastroenteropancreatic
HPF: high-power field
HR: hazard ratio
IRSD: index of relative socioeconomic disadvantage
NET: neuroendocrine tumor
OS: overall survival
WHO: World Health Organization
